# Growth of human breast tissues from patient cells in 3D hydrogel scaffolds

**DOI:** 10.1186/s13058-016-0677-5

**Published:** 2016-03-01

**Authors:** Ethan S. Sokol, Daniel H. Miller, Anne Breggia, Kevin C. Spencer, Lisa M. Arendt, Piyush B. Gupta

**Affiliations:** Whitehead Institute for Biomedical Research, 9 Cambridge Center, Cambridge, MA 02142 USA; Department of Biology, Massachusetts Institute of Technology, Cambridge, MA 02139 USA; Maine Medical Center Research Institute, Scarborough, ME 04074 USA; David H. Koch Institute for Integrative Cancer Research at MIT, Cambridge, MA 02139 USA; Department of Materials Science and Engineering, Massachusetts Institute of Technology, Cambridge, MA 02139 USA; Department of Comparative Biosciences, University of Wisconsin-Madison, Madison, WI 53711 USA; Harvard Stem Cell Institute, Cambridge, MA 02138 USA

## Abstract

**Background:**

Three-dimensional (3D) cultures have proven invaluable for expanding human tissues for basic research and clinical applications. In both contexts, 3D cultures are most useful when they (1) support the outgrowth of tissues from primary human cells that have not been immortalized through extensive culture or viral infection and (2) include defined, physiologically relevant components. Here we describe a 3D culture system with both of these properties that stimulates the outgrowth of morphologically complex and hormone-responsive mammary tissues from primary human breast epithelial cells.

**Methods:**

Primary human breast epithelial cells isolated from patient reduction mammoplasty tissues were seeded into 3D hydrogels. The hydrogel scaffolds were composed of extracellular proteins and carbohydrates present in human breast tissue and were cultured in serum-free medium containing only defined components. The physical properties of these hydrogels were determined using atomic force microscopy. Tissue growth was monitored over time using bright-field and fluorescence microscopy, and maturation was assessed using morphological metrics and by immunostaining for markers of stem cells and differentiated cell types. The hydrogel tissues were also studied by fabricating physical models from confocal images using a 3D printer.

**Results:**

When seeded into these 3D hydrogels, primary human breast epithelial cells rapidly self-organized in the absence of stromal cells and within 2 weeks expanded to form mature mammary tissues. The mature tissues contained luminal, basal, and stem cells in the correct topological orientation and also exhibited the complex ductal and lobular morphologies observed in the human breast. The expanded tissues became hollow when treated with estrogen and progesterone, and with the further addition of prolactin produced lipid droplets, indicating that they were responding to hormones. Ductal branching was initiated by clusters of cells expressing putative mammary stem cell markers, which subsequently localized to the leading edges of the tissue outgrowths. Ductal elongation was preceded by leader cells that protruded from the tips of ducts and engaged with the extracellular matrix.

**Conclusions:**

These 3D hydrogel scaffolds support the growth of complex mammary tissues from primary patient-derived cells. We anticipate that this culture system will empower future studies of human mammary gland development and biology.

**Electronic supplementary material:**

The online version of this article (doi:10.1186/s13058-016-0677-5) contains supplementary material, which is available to authorized users.

## Background

The ability to grow human tissues in three-dimensional (3D) cultures has proven useful, both for regenerative medicines and for studies of tissue development. Such “organoid” culture systems have been developed for several types of human tissues, including intestine, stomach, kidney, and brain [1–4]. For mammary tissue, collagen matrices were first introduced four decades ago for growing mammary spheroids from primary mouse epithelial cells [5, 6]. Subsequently, Barcellos-Hoff and colleagues developed a basement membrane (Matrigel) culture in which mouse epithelial cells generated ducts and lobules, enabling the first studies of mammary morphogenesis in vitro [7].

While these and similar 3D cultures have contributed valuable insights [8–13], the biology of mouse mammary tissue is known to differ in significant ways from its human counterpart [14, 15]. To address this issue, investigators have developed 3D cultures that support organoid growth from human cell lines that have been immortalized by transduction with viral oncogenes [16–18]. However, growing tissues from primary human mammary cells has proven to be more challenging. Tanos and colleagues maintained viable primary human mammary tissue fragments in liquid cultures for up to 6 days [19], but their cultures did not support ductal initiation or elongation. Ductal growth was also limited in 3D cultures of primary human cells seeded into collagen or basement membrane (Matrigel) [20, 21].

The extracellular matrix (ECM) plays a critical role in regulating the development and maintenance of epithelial tissues. The ECM of human breast tissue is a complex mixture of protein fibrils interwoven within a network of glycosaminoglycan carbohydrate chains. From a structural perspective, the protein components, including laminins, fibronectin, and collagens, provide resistance to tensile forces, while the carbohydrates—composed primarily of hyaluronan chains—chelate water and provide resistance to compressive forces.

To more fully reflect this complexity, we engineered a hydrogel scaffold that incorporated both the protein (collagen, laminins, and fibronectin) and carbohydrate components (hyaluronan) of human breast tissue. When seeded into these hydrogels, primary mammary epithelial cells isolated from patient breast tissues self-organized, expanded, and differentiated to form mature mammary tissues. We anticipate that these cultures will prove useful in future investigations of human mammary tissue morphogenesis and biology.

## Methods

### Ethics statement

Primary tissues that would otherwise have been discarded as medical waste following surgery were obtained in compliance with all relevant laws, using protocols approved by the institutional review board at Maine Medical Center. All tissues were anonymized before transfer and could not be traced to specific patients; for this reason, this research was provided exemption status by the Committee on the Use of Humans as Experimental Subjects at the Massachusetts Institute of Technology. All patients enrolled in this study signed an informed consent form to agree to participate in this study and for publication of the results.

### Preparation of primary patient-derived tissue

Reduction mammoplasty tissue samples were mechanically dissociated and then incubated with 3 mg/ml collagenase (Roche Life Science, Indianapolis, IN, USA) and 0.7 mg/ml hyaluronidase (Sigma-Aldrich, St. Louis, MO, USA) at 37 °C overnight. Epithelial clusters were disrupted by trituration, washed, and depleted for fibroblasts. The identical procedure was used to prepare mouse mammary epithelial tissues.

### Preparation of hydrogels

Hydrogels were composed of 1.7 mg/ml collagen I (Corning), 10 μg/ml hyaluronan 150 and 500 kDa (Sigma-Aldrich), 40 μg/ml laminin isolated from Engelbreth-Holm-Swarm sarcoma cells (Life Technologies, Grand Island, NY, USA), and 20 μg/ml fibronectin (Life Technologies), pH 7.3, to which tissue fragments and growth factors were added (for details, see Additional file 1: Supporting Methods). Hydrogels were produced in a four-chamber slide (Corning, Corning, NY, USA) as a mold and incubated at 37 °C for polymerization. These gels partially polymerized within 5 minutes and fully solidified within 1 h, at which time they were detached from the mold. Structures were passaged from one hydrogel to another by dissolving the pad with collagenase and reseeding the structure as if it were a primary tissue fragment. All experiments were performed with at least four independent replicates (*n*) using samples from at least three patients (*k*), unless otherwise specified (*N* = *n*, k).

### Lentivirus production

Lentivirus production was performed as previously described [22]. Lentiviral gene ontology vectors were kindly provided by Kristoffer Riecken [23].

### Immunofluorescence and immunohistochemistry

Immunofluorescence was performed as previously described [24]. Immunohistochemical staining was performed at the Koch Institute Histology Core using the Thermo Scientific IHC Autostainer 360 (Thermo Scientific, Waltham, MA, USA).

### Microscopy

Images were captured using a Zeiss LSM 700 (immunofluorescence; Zeiss Microscopy, Thornwood, NY, USA), a Zeiss Axiophot (immunohistochemistry; Zeiss Microscopy), and a Nikon TE2000 (Nikon Instruments, Melville, NY, USA) with a heated stage and 5 % CO_2_ (time lapse).

## Results

### Design of hydrogels with features of human breast tissue

Because we were interested in engineering a 3D scaffold that could stimulate the growth of human breast tissues, we explored hydrogel formulations that contained protein and glycosaminoglycan components found in the ECM of human breast tissue. We focused our efforts on ECM hydrogels with defined components and evaluated various hydrogel formulations by assessing their ability to support the growth of primary human breast tissue fragments. The seeded tissue fragments contained 50–100 cells per fragment and were harvested by dissociating breast tissues from patient reduction mammoplasties (Additional file 2: Figure S1).

**Fig. 1:**
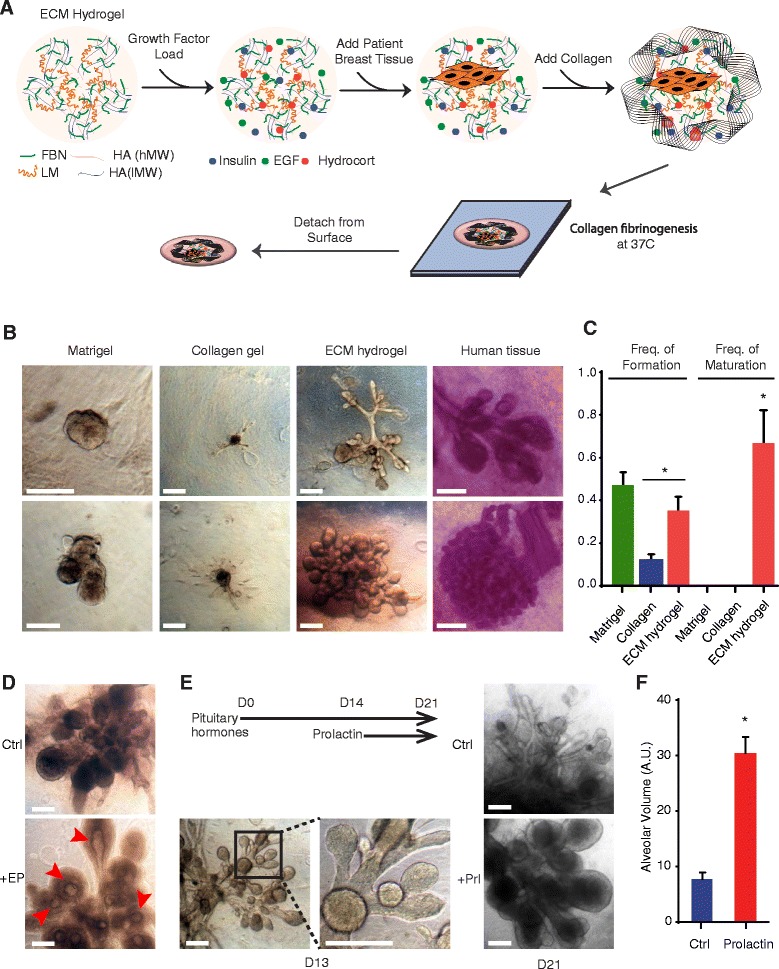
Extracellular matrix (ECM) hydrogels enable self-organization and hormone responsiveness of human breast organoids. **a** Schematic representation of hydrogel assembly. Fibronectin, FBN; laminin, LM; high molecular weight hyaluronan, HA(hMW); low molecular weight hyaluronan, HA(lMW); epidermal growth factor, EGF. **b** Comparative morphology of human breast tissue in vivo and ex vivo. Representative bright-field images of ex vivo organoid growths after 10 days in Matrigel, polymerized collagen, or ECM hydrogels next to representative bright-field whole-mount images of carmine-stained human breast tissue. **c** Quantification of outgrowths in Matrigel, collagen, or ECM hydrogels. Frequency of seeded tissue fragments expanding (Freq. Formation) and frequency of expanded outgrowths exhibiting TDLU architecture (Freq. Maturation) from a patient. **d** Bright-field images of organoid growth after 3 weeks with or without estrogen and progesterone (+EP) treatment. Maturation of ducts and lobules is evident by hollowed lumens (*red arrowheads*). **e** Experimental design and representative bright-field images of organoid outgrowths in ECM hydrogels in response to pituitary hormones and recombinant human prolactin (Prl). Bovine pituitary extract was added at seeding (D0) and prolactin at day 14 (D14) (*n* = 4, 7, and 14, 1 resp). *Bottom left* images (D13) show representative morphology before treatment with prolactin (D21). **f** Quantification of lobular volume (arbitrary units, AU) with or without prolactin treatment described in (**e**). Error bars represent standard error of the mean. Scale bars represent 200 μm. *p < 0.01

Through heuristic optimization, we established a novel hydrogel formulation that supported the growth of human breast tissues (Fig. 1a). These hydrogels had several features that were important for supporting breast tissue growth: (1) They were fabricated with collagen, fibronectin, and laminin, three ECM proteins present in human breast tissue in vivo [25]; (2) they incorporated hyaluronic acid, a glycosaminoglycan polysaccharide present in many human tissues, including the breast; (3) they were loaded with three growth factors (insulin, epidermal growth factor, and hydrocortisone) that support the growth and differentiation of mammary epithelial cells [21, 26, 27]; and (4) they were cultured in suspension [6, 28, 29].

To assess their physical properties, we compared the swelling ratio and elasticity (Young’s modulus) of the ECM hydrogels with those of gels consisting only of collagen. The ECM hydrogels exhibited a significantly higher swelling ratio than collagen gels (306.94 ± 6.29 vs 290.10 ± 0.81, *p* < 0.01). This difference was likely due to inclusion in the ECM hydrogels of hyaluronans, which bind and retain water. Atomic force microscopy (AFM) indicated that the ECM hydrogels had significantly increased elasticity relative to collagen gels (Young’s modulus of 256.7 ± 20.0 Pa vs. 559.2 ± 204.0 Pa, *p* < 0.05) (Additional file 2: Figure S2). One potential explanation for this difference is that the additional ECM components in the hydrogels could be partially disrupting the efficiency of collagen polymerization, thereby resulting in a more elastic hydrogel that more closely approximated the elasticity of breast tissue in vivo [30]. These findings indicated that the ECM hydrogels were more elastic with increased water content relative to collagen-only gels.

### ECM hydrogels support the growth of complex breast tissues

When seeded into the ECM hydrogels, primary mammary epithelial cell clusters isolated from reduction mammoplasties rapidly grew into complex breast tissues with a seeding efficiency of approximately 33 % (Fig. 1b, c). The majority of breast tissues that expanded in the hydrogels had complex ductal and lobular morphologies that closely resembled the epithelial structures present in the human breast (67 %) (Fig. 1b). The expanded breast tissues exhibited similar morphologies across all seven of the patient samples that were assessed. In contrast, and consistent with prior findings [31], there was minimal or no outgrowth when primary mammary cells were seeded either into collagen-only gels or into basement membrane with or without additional ECM components (Fig. 1b and c; Additional file 2: Figure S3a). The rare outgrowths that formed in collagen-only gels were either thin ducts or spheres, whereas the outgrowths in basement membrane were spheres with some ruffling at the edges.

The efficiency of tissue formation was much lower when single primary epithelial cells were seeded into the hydrogels (0.16 %), when compared with the efficiency observed with primary cell clusters (33 %). Moreover, only 4.5 % of the organoids derived from single cells exhibited the complex ductal and lobular morphologies that were exhibited by the majority of tissues grown from primary cell clusters. Significantly, the few single-cell–derived organoids with complex morphologies contained only cytokeratin 14 (CK14)-positive basal cells and did not contain CK8/18-positive luminal cells (Additional file 2: Figure S4). On an absolute scale, 0.0075 % of single cells gave rise to tissues with complex ductal and lobular morphologies, whereas 26 % of primary cell clusters gave rise to tissues with complex ductal and lobular morphologies (Additional file 2: Figure S4). The structures that formed from single cells were primarily thin ducts (83.6 %) and, less frequently, simple lobules (11.9 %). These findings indicated that single cells can form topologically complex structures at a low frequency, but the resulting structures contained only one of the two major cell lineages present in the mammary gland in vivo and in the tissues grown from primary cell clusters. In light of these observations, we focused subsequent experiments on growing tissues from primary cell clusters.

### Tissues exhibit morphological response to hormones

We next assessed if the human mammary tissues grown in hydrogels responded to steroid, pituitary, and lactogenic hormones, which are known to stimulate the development of mammary epithelial tissue in vivo*.* Staining the hydrogel tissues for hormone receptors revealed that approximately 5 % of the cells expressed each of the estrogen and progesterone receptors (ER and PR, respectively) (Additional file 2: Figure S5). Treatment of the hydrogels with estrogen and progesterone stimulated the mammary tissues to hollow, resulting in the formation of ducts and lobules with evident lumens (Fig. 1d, Additional file 2: Figure S5). This suggested that estrogen and progesterone were promoting further maturation of the mammary tissues.

Extracts of the pituitary gland contain factors important for mammary development, including growth hormone, fibroblast growth factors, and follicle-stimulating hormone [32, 33]. Consistent with this, addition of pituitary extracts to the ECM hydrogels caused a significant increase in both secondary and tertiary ductal branching of the expanded breast tissues (Fig. 1e). Moreover, addition of both pituitary extract and prolactin further stimulated lobular expansion with a fourfold increase in lobular volume accompanied by the formation of large lipid droplets that were visible upon hematoxylin and eosin staining (Fig. 1e and f, Additional file 2: Figure S5).

### Kinetics of tissue growth and maturation in hydrogels

**Fig. 2:**
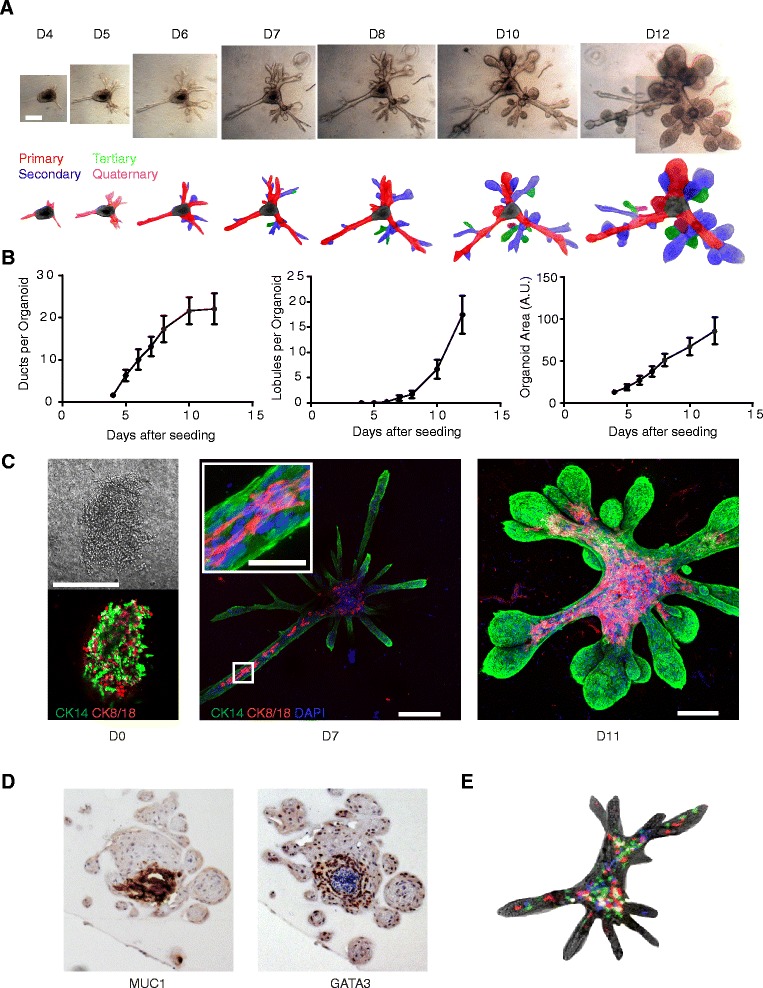
Human breast tissue undergoes morphogenesis and differentiation in hydrogels. **a** Bright-field images (*top*) and schematic representation (*bottom*) of the dramatic expansion and maturation of organoids over the course of 12 days. Scale bar represents 200 μm. **b** Quantification of the number of ducts per organoid, lobules per organoid, and cross-sectional area of organoids during a 12-day time course (*N* = 9 organoids). **c** Immunofluorescence of luminal CK8/18 (*red*) and myoepithelial CK14 (*green*) marker expression at seeding (*left*), 7 days (*middle*), or 11 days (*right*) after seeding. Upon seeding cells are disorganized, but they self-organize into bilayered organoids. By 11 days after seeding, outgrowths had matured and the CK8/18^+^ cells fully lined the luminal layer, while the CK14^+^ cells were basally localized. Nuclei were stained with DAPI (*blue*). *Inset* scale bar represents 50 μm. All other scale bars represent 200 μm. Also see Additional file 2: Figure S7 and Additional file 3: Movie S1. **d** Immunohistochemical staining of MUC1 and GATA3 expression in organoids after 21 days of culture. Representative image from one patient is shown. **e** Bright-field and fluorescence overlaid image of an organoid outgrowth following infection of cells with multicolored lentivirus. Clonal tracking shows heterogeneous localization of clones during organoid morphogenesis and ductal elongation. Also see Additional file 4: Movie S2, Additional file 5: Movie S3, Additional file 6: Movie S4, and Additional file 7: Movie S5. Error bars represent standard error of the mean. *A.U.* arbitrary units, *DAPI* 4′,6-diamidino-2-phenylindole, *CK* cytokeratin

To examine the kinetics with which these tissues matured, we captured bright-field images over a span of 8 days, beginning at the earliest time point at which we observed ductal outgrowths (day 4) (Fig. 2a and Additional file 2: Figure S6). These primary ductal outgrowths gave rise to secondary and tertiary ducts over the next week, either through bifurcation of elongating ducts or through side branches that sprouted from ducts. After 8–12 days of tissue growth, there was a rapid increase in the number and size of lobules (Fig. 2a and b).

The primary cells seeded into hydrogels were initially disorganized clusters with intermixed basal (CK14^+^) and luminal (CK8/18^+^) cells (Fig. 2c). However, by 7 days, the cells had self-organized into an outer CK14^+^ basal layer with some CK8/18^+^ luminal cells in the interior of the expanding tissues (Fig. 2c, *center*, Additional file 2: Figure S7A, Additional file 3: Movie S1). At this early time point, the majority of newly initiated ducts were small and exclusively composed of CK14^+^ basal cells. However, as the organoids expand and mature, CK8/18^+^ luminal cells can be seen lining their interior (Fig. 2c, *right*, Additional file 2: Figure S7B). In all patients, at least 60 % of the mature organoids contained distinct luminal and basal layers.

By 21 days, there was clear evidence of tissue maturation, with the lobule interiors staining strongly for both the luminal lineage marker GATA3 and the luminal differentiation marker MUC1 (Fig. 2d). At this time, some of the lobule interiors also showed evidence of cavitation (Fig. 2d). Fully mature structures expanded to sizes of up to 3 mm in diameter (Additional file 2: Figure S8) and remained viable for at least 8 weeks in culture in the same hydrogel. During this time, the developing and expanding tissues radically remodeled and condensed the hydrogels in which they were cultured, with evidence of this condensation up to 2 mm away (Fig. 2a and Additional file 2: Figure S6, S9). After 3–6 weeks, the organoids fully expanded to the size of the condensed pad and were unable to grow further. However, these structures could be removed from the hydrogels by enzymatic digestion and reseeded into new hydrogels, which support their continued growth (Additional file 2: Figure S3B).

Prior studies of the morphogenesis of mouse mammary organoids have indicated that the process of ductal initiation and elongation involves a dynamic reorganization of cells within 3D cultures [9]. To assess if this was also occurring in our primary human organoids, we stably labeled the primary cell clusters with fluorescent proteins before seeding them into the hydrogel scaffolds. Because the fluorescent proteins were delivered by lentivirus at a low multiplicity of infection, it was possible to assess the contributions of individual clones and their progeny to the formed mammary tissues. Using this approach, we found that the progeny of individual clones were dispersed throughout the tissue structures rather than being localized to clonal patches (Fig. 2e). This suggested that cells underwent dynamic rearrangements as they proliferated to grow tissues. Time-lapse movies also showed dynamic rearrangements. Mass cell migrations could be seen in the organoid cores, along ducts, and also within terminal ductal lobular units (TDLUs) (Additional file 4: Movie S2, Additional file 5: Movie S3, and Additional file 6: Movie S4).

### Mammary stem cell behavior in ductal initiation and maturation

**Fig. 3:**
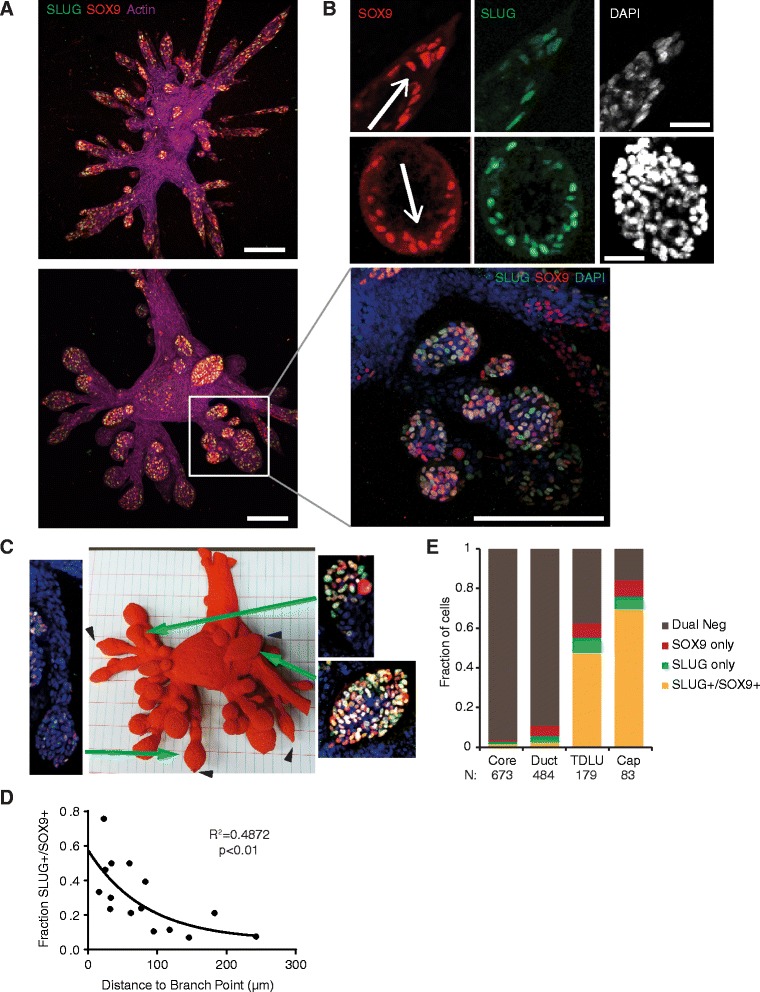
Mammary stem cells (MaSCs) are enriched and localized to the leading edge of elongating outgrowths and side branches. **a** Two representative immunofluorescence images of SLUG (*green*), SOX9 (*red*), and actin (*purple*) expression in day 10 outgrowths. Scale bar represents 200 μm. **b** *Top*: Immunofluorescence of SLUG, SOX9, and DAPI at the growing tips of ducts. *Arrows* indicate the direction of growth. Scale bars represent 50 μm. *Bottom*: High-magnification images reveal that new side branches are enriched for SLUG^+^/SOX9^+^ double-positive cells. Scale bar represents 200 μm. **c** Photomicrograph of the 3D printed organoid outgrowth from (**a**). Leader cells could be visualized and located at the leading edge of many outgrowths (*black arrowheads*). Immunofluorescence images depict SLUG (*green*), SOX9 (*red*), and DAPI (*blue*) in representative outgrowths with long (*left*), intermediate (*top right*), and short (*bottom right*) ductal elongation. **d** Quantification of the number of SLUG^+^/SOX9^+^ cells in relation to duct length revealed a significant anticorrelation between the number of double-positive cells and duct length (*p* < 0.01). **e** Quantification of the fraction of SLUG^+^ and SOX9^+^ cells in the indicated regions of the organoid outgrowths in (**a**). Every pairwise comparison was statistically significant (*p* < 0.001). *N* indicates the number of cells quantified.

To identify putative mammary stem cells (MaSCs), we performed immunofluorescence staining for the transcription factors SLUG and SOX9, which, when coexpressed, mark MaSCs in the murine mammary gland [34]. SLUG^+^/SOX9^+^ cells were rarely seen within the core and ducts of organoids, but they made up roughly half of the cells in the TDLUs. These TDLUs were typically five to eight cells thick, and the layer of cells in direct contact with the ECM (termed the *cap region*) was most enriched for the dual-positive cells, with roughly two-thirds of cells coexpressing SLUG and SOX9 (Fig. 3a, e). In both ducts and lobules, the dual-positive cells were enriched in the cap region of the expanding outgrowth, in direct contact with the ECM, suggesting that this contact could be involved in maintaining stem cells in an undifferentiated state (Fig. 3b, e).

To assess the topological properties of these organoids, we rendered a surface model from 3D confocal microscopic images and used a Dimension Elite 3D printer (Stratasys, Eden Prairie, MN, USA) to fabricate a high-resolution 1500X scale physical model of an organoid stained for filamentous actin (Fig. 3c). Examination of this physical model revealed that the outgrowths containing the highest fraction of SLUG^+^/SOX9^+^ cells were also the shortest. When side-branches started to form, nearly all of the cells were dual-positive, but as the ducts elongated, there was a gradual decrease in the fraction of dual-positive cells (Fig. 3d). This suggested that side branches were initiated by the proliferation of SLUG^+^/SOX9^+^ cells, which subsequently differentiated to give rise to interior cells concurrently with ductal elongation.

### SLUG^+^/SOX9^+^ leader cells direct ductal elongation

**Fig. 4:**
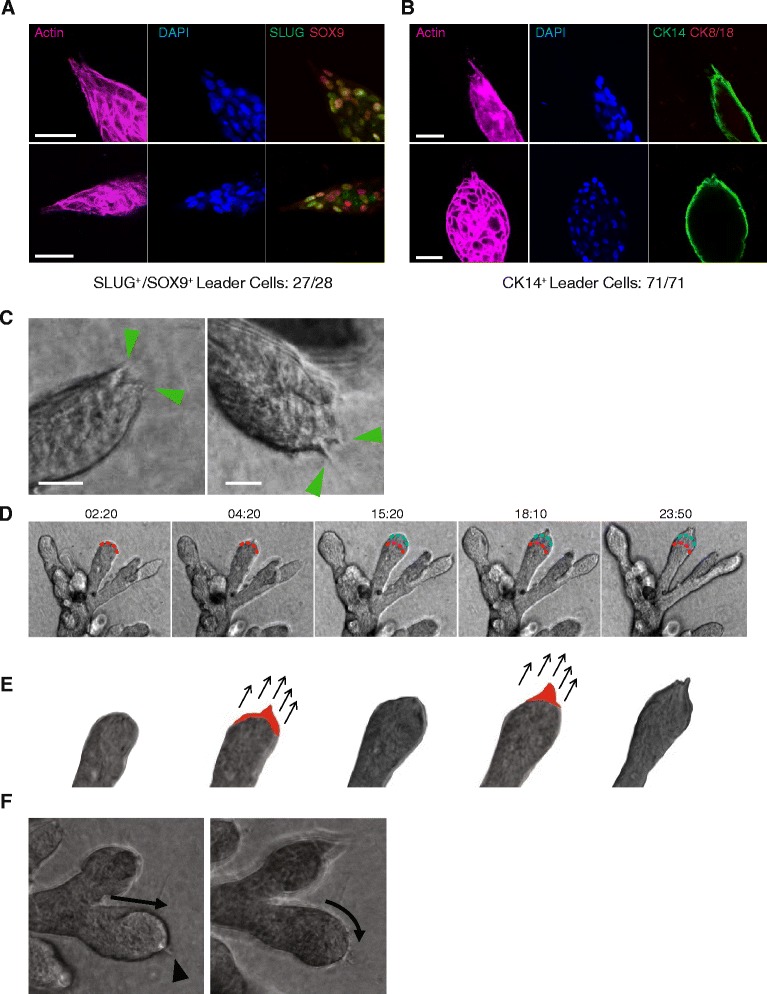
Leader cells drive tissue morphogenesis in hydrogels. **a** Expression of SLUG and SOX9 in leader cells. Fluorescence image of actin-rich terminal ductal lobular unit (TDLU) protrusions stained with phalloidin (*purple*), α-SLUG (*green*), and α-SOX9 (*red*). Nuclei stained with 4′,6-diamidino-2-phenylindole (DAPI; *blue*) and quantification of double-positive leader cells (*yellow*) indicated below. Scale bars represent 50 μm. **b** Expression of cytokeratin 14 (CK14) and CK8/18 in TDLU protrusions. Fluorescence images of actin-rich protrusions stained with phalloidin (*purple*), α-CK14 (*green*), and α-CK8/18 (*red*). Nuclei stained with DAPI (*blue*) and quantification of CK14 positivity in leader cells indicated below. Scale bars represent 50 μm. **c** Bright-field images of TDLU protrusions containing multiple divergent leader cells (*green arrowheads*). **d** Time-lapse microscopy of a day 8 TDLU showing a single leader cell protruding at the leading ductal edge (*red line*), followed by ductal elongation (*blue line*) and the eventual appearance of another leader cell at the new leading edge. See Additional file 6: Movie S4 and Additional file 7: Movie S5. Time denotes hours:minutes following onset of recording. **e** Schematic depiction of the single duct elongating in (**d**), highlighting in *red* the protruding leader cell that precedes ductal elongation. *Arrows* indicate the direction of invasion and elongation. **f** Time-lapse analysis of an 8-day TDLU that changed its direction during ductal elongation. A single leader cell (*black arrowhead*) appears to reorient the direction of ductal growth (*arrows*). See Additional file 8: Movie S6. Scale bars represent 50 μm

Examination of the printed 3D model also revealed the presence of small tips at the leading edges of elongating ducts. Confocal microscopy showed that these tips contained one or two leader cells that were polarized in the direction of ductal elongation. The leader cells stained positively for filamentous actin and protruded from the structures in the direction of ductal elongation (Fig. 4a and b). The leader cells expressed basal cytokeratins (Fig. 4b) and coexpressed SLUG and SOX9 (Fig. 4a). While the majority of outgrowths contained one leader cell, occasionally outgrowths contained multiple leader cells in different orientations (Fig. 4c).

Time-lapse microscopy provided additional insights into the relationship between these leader cells and ductal elongation. Ductal elongation was always preceded by a transient extension of leader cells that physically engaged with and deformed the ECM (Fig. 4d and e, Additional file 4: Movie S2, Additional file 7: Movie S5). At times, the force of this interaction between leader cells and the matrix caused them to break away from the ducts and become isolated in the matrix (Additional file 4: Movie S2). The direction in which the leader cells extended was always the direction of the next wave of ductal elongation. When the direction in which the leader cells emanated was different from the previous direction of elongation, the ducts reoriented in the new direction specified by the leader cells before the next wave of elongation (Fig. 4f, Additional file 8: Movie S6). This ductal reorientation appeared to be induced by the collective rotation of cells in the lobule, which occurred before ductal elongation (Additional file 8: Movie S6). After the ducts reoriented, they elongated for a period of time, after which the elongation ceased. After ductal elongation ceased, new leader cells emanated from the ductal tips to initiate the next cycle of elongation.

A prior study of murine mammary organogenesis indicated that ductal elongation is driven not by leader cells but rather through the collective expansion and migration of luminal cells [9]. Because this prior study was conducted using Matrigel, the discrepancy with our observations could be due either to differences in the types of 3D scaffolds used or to differences in biology between mouse and human mammary cells. When mammary tissue fragments from C57BL/6 J mice were seeded into our ECM hydrogels, they grew and ruffled but did not exhibit any leader cell activity (Additional file 2: Figure S3). This finding suggested that leader cells may play a role specifically in the morphogenesis of human mammary tissue and not that of mice.

## Discussion and conclusions

Our findings demonstrate the possibility of growing human mammary tissues from patient-derived cells in ECM hydrogels containing only defined and physiologically relevant components. The tissues that form in these hydrogels consist of multiple cell lineages and respond to steroid, pituitary, and lactogenic hormones. While stromal cells are essential for making ECM in vivo, these findings indicate that their active and continued participation is not essential for the morphogenesis and growth of human mammary tissue. Although somewhat unexpected, given the instructive role that stromal cells appear to play in the developing mammary gland in vivo [35], this finding is consistent with observations that have been made in other organoid systems. For example, intestinal epithelial cells self-organize into intestinal crypts when placed into basement membrane cultures [3], lingual epithelial cells recapitulate the complex organization of tongue epithelium [36], and neuroectodermal cells self-organize into cerebral organoids that recapitulate key aspects of brain development [1]. An emerging theme derived from these studies is that epithelial cells have an inherent ability to self-organize into complex tissues without the support of stromal cells, provided they are placed into suitable 3D culture conditions.

Because our breast tissues were cultured in transparent hydrogels, we were able to directly observe the processes of ductal initiation, elongation, and branching. We observed two main methods of branching, both previously seen in mouse mammary morphogenesis [37, 38]: (1) bifurcation at the ends of ducts and (2) ductal side branching. Interestingly, we found that ductal budding and elongation in the primary human tissues was driven by SLUG^+^/SOX9^+^ leader cells that express filamentous actin and basal cytokeratins (CK14^+^). Leader cells do not appear to play a role in ductal elongation in mouse mammary organoids, which is instead driven by the mass action of luminal cell layers [9]. However, leader cells with filamentous actin-positive protrusions have been implicated in ductal elongation in the air sacs of flies [38, 39] and in vascular endothelia [40]. Taken together with results of previous studies [9], our findings suggest that different species may use very different mechanisms to promote mammary morphogenesis.

We were able to identify where putative human MaSCs were localized by staining for SLUG and SOX9, which label MaSCs in mice. Cells that were dual-positive for these markers were localized primarily to the cap regions of new outgrowths and were in direct contact with the ECM. This finding raises the possibility that ECM contact may be necessary to maintain stem cells in an undifferentiated state. This is consistent with the role of ECM in regulating stem cell self-renewal in the hematopoietic system, hair follicles, and the brain [41].

The localization of stem cells to the tips of developing lobules is consistent with recent findings in the human breast [42]. By sectioning and staining primary human tissue, Honeth et al. found that MaSCs were enriched at the tips of immature lobules, with decreased MaSC numbers in larger and more mature lobules. The possibility that MaSCs may be localized to the cap region of end buds has also been proposed for the murine mammary gland [43, 44].

We found that the SLUG^+^/SOX9^+^ leader cells are motile and express the basal cytokeratin CK14. These findings are consistent with prior studies demonstrating that MaSCs are found in the basal cell compartment [45–47], as well as reports that induction of mammary cells into a stem-like state results in the upregulation of basal markers and an onset of motility [48]. This raises the possibility that the cells in, or induced into, a stem cell state are simultaneously capable of self-renewal and capable of maneuvering through and engaging the ECM. These programs could be coopted by cancer cells, where the properties of self-renewal (allowing for continued proliferative potential) and motility through the ECM (allowing for dissemination and expansion) might be selected for. We anticipate that the ability to grow hormone-responsive human breast tissue in hydrogels with defined components will empower future studies of human mammary gland development and biology, with potential implications for the understanding of breast cancer biology.

Linnemann et al. recently reported alternative 3D culture conditions for the expansion of TDLU-like structures from primary human cells [49]. While their conditions have the advantage of supporting the growth of single cells at high efficiency, this comes with the drawback of incorporating serum and chemical agents (Rho-associated protein kinase inhibitor, forskolin) that perturb intracellular signaling in nonphysiological ways. By contrast, although our culture system can only grow tissues at high efficiency from clusters of cells, it has the advantage of incorporating only defined components that are physiologically relevant, which, among other things, makes it possible to study how hormones impact tissue morphogenesis and differentiation. These relative merits will need to be carefully considered when deciding which system to use in future studies.

## Additional files

Additional file 1:
**Supporting Methods [** 22 **,** 23 **,** 50 **].** (DOC 66 kb) Additional file 2: Supplemental Figures.
**Figure S1** Schematic for the dissociation of reduction mammoplasty tissue. Schematic representation of tissue dissociation and purification of epithelium. For more details, see Additional file 1: Supporting Methods. **Figure S2** Physical characterization of collagen and ECM hydrogels. **a** Young’s modulus was measured at least three times for each of three independent replicates (*red*, *blue*, and *green*) for collagen gels and ECM hydrogels. Plotted are the mean and standard deviation for the replicates. **b** The swelling ratio was calculated for collagen gels and ECM hydrogels for four independent replicates. Plotted are the mean and standard deviation. **p* < 0.05. **Figure S3** Comparison of various 3D scaffolds seeded with mouse or human mammary tissue. Representative bright-field images of human or mouse mammary epithelial tissue fragments grown for 10 days in either Matrigel alone (Matrigel); Matrigel supplemented with fibronectin, laminins, hyaluronans, insulin, epidermal growth factor, and hydrocortisone (Matrigel + ECM); collagen hydrogels (collagen gel); or collagen hydrogels supplemented with fibronectin, laminins, hyaluronans, insulin, EGF, and hydrocortisone (ECM hydrogel). **b** Representative bright-field images of an organoid grown in an ECM hydrogel (*left*), removed from the primary gel using collagenase treatment and fragmented (*middle*), and producing new outgrowths after being passaged into a secondary ECM hydrogel (*right*). Scale bars represent 200 μm. **Figure S4** Single mammary epithelial cells produce heterogeneous structure morphologies in hydrogels. Representative bright-field and immunofluorescence images of structures formed from single cells after 18 days of growth in hydrogels. These structures are highly heterogeneous but generally fall into three classes: ductal structures with narrow ducts and no lobules (*top*), lobular structures with short and wide ducts (*middle*), and structures with mixed ductal and lobular architecture (*bottom*). The majority of structures formed from single cells are either exclusively ductal or exclusively lobular, with only 4.5 % of structures scored showing mixed architecture. Immunofluorescence staining for luminal and basal cytokeratins demonstrates that even mixed-architecture organoids derived from single cells do not contain both mature cell types, as CK8/18 staining was never observed. Scale bars represent 200 μm. **Figure S5** Organoids respond to hormone treatment. **a** Hematoxylin and eosin staining of organoids treated with either vehicle or prolactin (1 μg/ml). Lipid droplets can be seen following prolactin treatment. **b** *Left*: Confocal maximum intensity projection of an organoid grown for 3 weeks in estrogen (10 ng/ml) and progesterone (500 ng/ml). *Right*: Serial sections through the organoid shows hollow ducts and lobules. Distance of the section from the surface of the structure is indicated. Scale bars represent 200 μm. **c** Immunohistochemical staining of day 5 organoids grown in the presence of estrogen (10 ng/ml) and progesterone (500 ng/ml). *Left*: Progesterone receptor staining. *Right*: Estrogen receptor staining. *Arrowheads* indicate a subset of positive cells. Scale bars represent 100 μm. **Figure S6** Expansion and maturation of organoids. Bright-field microscopy demonstrates massive expansion and maturation of organoids, with lobule formation initiating after day 5 and maturation of TDLUs by day 12. Note that, by day 12, the surrounding ECM has been dramatically condensed, leading to reduced visibility. Scale bars represent 500 μm. **Figure S7** Organoids self-organize and differentiate. **a** Immunofluorescence microscopy shows that a myoepithelial layer (CK14, *green*) completely surrounds the exterior of an organoid after 7 days of growth in hydrogel, while a luminal layer (CK8/18, *red*) forms in the interior. Note that smaller outgrowths from the central core are exclusively myoepithelial, while larger, more mature outgrowths contain a luminal layer. **b** Organoids after 14 days of growth in hydrogels. Scale bars represent 200 μm. **Figure S8** Organoids are capable of growing up to 3 mm in diameter. **a** Confocal microscopy of actin (phalloidin, *red*) and nuclear (DAPI, *blue*) staining of an organoid grown for 3 weeks in a hydrogel. Scale bar represents 2 mm. **b** Bright-field imaging of organoids grown for 2 weeks (*left*) and 4 weeks (*right*) in hydrogels. Scale bars represent 1 mm. **Figure S9** Organoids perform long-range ECM remodeling. **a** Bright-field image of organoids grown in a hydrogel for 4 weeks. Note the condensed ECM spanning the distance between the two large organoids in a noncellular region of the hydrogel. Contrast was increased to better distinguish condensed ECM from uncondensed ECM. Scale bar represents 1 cm. **b** Time-course bright-field microscopy shows that organoids seeded into a hydrogel at an initial distance of roughly 1 mm apart align their outgrowths to grow toward one another, indicating long-range communication through the hydrogel. By day 12 of growth in the hydrogel (*bottom*), the three organoids have fused together. Scale bars represent 0.5 mm. (PDF 13277 kb) Additional file 3: Movie S1. Confocal Z-series panning through the duct depicted in Fig. 2c (*right*). Z fields are separated by 1.11 μm, spanning a total of 71.94 μm. Note that the exterior of the structure is completely encapsulated by the myoepithelial layer of cells (CK14, *green*) and that the luminal layer is restricted to the inner layer of cells (CK8/18, *red*). (AVI 202753 kb) Additional file 4: Movie S2. Time-lapse microscopy depicting a rotating lobule and the behavior of leader cells. The *arrow* indicates the direction of rotation of the lobule on the *left. Arrowheads* indicate the location of leader cells extending from two elongating outgrowths. At the outset of the movie, the organoid had been grown for 8 days. Images were captured every 10 minutes. Frame rate: eight frames per second. (AVI 9677 kb) Additional file 5: Movie S3. Time-lapse microscopy depicting dynamic cellular migration in a growing organoid. The *arrows* indicate the direction of mass migration along two elongating ducts from the core of the organoid toward the leading edge of the ducts. At the outset of the movie, the organoid had been grown for 8 days. Images were captured every 10 minutes. Frame rate: eight frames per second. (AVI 5319 kb) Additional file 6: Movie S4. Time-lapse microscopy depicting dynamic cellular migration in a growing organoid. The *arrows* indicate the direction of mass migration away from the leading edge of one duct and into another elongating duct. At the outset of the movie, the organoid had been grown for 8 days. Images were captured every 10 minutes. Frame rate: eight frames per second. (AVI 7540 kb) Additional file 7: Movie S5. Twenty-four-hour time-lapse movie of an organoid growing in hydrogel. Still frames from this movie are depicted in Fig. 4d. At the outset of the movie, the organoid had been grown for 8 days. Images were captured every 10 minutes. Frame rate: eight frames per second. (AVI 19903 kb) Additional file 8: Movie S6. Time-lapse microscopy depicting a leader cell redirecting the orientation of elongation of an outgrowth. At the outset of the movie, the organoid had been grown for 8 days. Images were captured every 10 minutes. Frame rate: eight frames per second. (AVI 15328 kb)

## Abbreviations

EGF: epidermal growth factor
FBN: fibronectin
HA: hyaluronan
hMW: high molecular weight
LM: laminin
lMW: low molecular weight
AFM: atomic force microscopy
A.U.: arbitrary units
BrdU: bromodeoxyuridine
CK: cytokeratin
DAPI: 4′,6-diamidino-2-phenylindole
ECM: extracellular matrix
ER: estrogen receptor
MaSC: mammary stem cell
PR: progesterone receptor
Prl: prolactin
TDLU: terminal ductal lobular unit
3D: three-dimensional
