# Supplementary material for: Growth of human breast tissues from patient cells in 3D hydrogel scaffolds
Source: Breast Cancer Res. 2016 Mar 1;18:19. doi: 10.1186/s13058-016-0677-5 (PMC4772689; doi:10.1186/s13058-016-0677-5)
Supplement: Additional file 1: — Supporting Methods [ 22 , 23 , 50 ]. (DOC 66 kb) [file 13058_2016_677_MOESM1_ESM.doc]

**Supporting Methods**

***Processing of Primary Breast Tissues***

Breast tissues from patient reduction mammoplasties were obtained from the Maine Medical Center Biobank. The breast tissues were typically 20 – 100 grams, which was enough material to seed ~200-800 mL of ECM hydrogels. Mouse mammary tissue was collected from 12-week old C57BL/6 mice. Tissues were mechanically dissociated using a sterile razor blade into approximately 3-5 mm^3^ fragments, and resuspended in Dissociation Buffer (MEGM (Lonza) containing 3 mg/mL collagenase (Roche), 250 units/mL hyaluronidase (Sigma Aldrich), 1X Anti-anti (Gibco)) at a concentration of 0.2 gm/mL, and incubated with rocking at 37 C overnight. Organoids were allowed to pellet by gravity for five minutes, and were washed five times in PBS containing 5% FBS (Sigma), in order to remove any associated stromal cells. Prior to seeding into hydrogels, further fibroblast depletion was carried out by plating organoids in DMEM containing 10% FBS on tissue culture treated dishes for 90 minutes. Organoids were then washed in PBS and resuspended in culture media.

***Preparation of hydrogels***

Hydrogels were composed of 1.7 mg/mL rat tail collagen (EMD Millipore), 10 µg/mL hyaluronan 150 kDa (Sigma Aldrich), 10 µg/mL hyaluronan 500 kDa (Sigma Aldrich), 40 µg/mL laminin (Life Technologies), and 20 µg/mL fibronectin (Life Technologies), supplemented with 0.05% insulin, 0.05% hydrocortisone, and 0.05% epidermal growth factor (Lonza CC-4021G, CC-4031G, and CC-4017G respectively). Collagen was first pH neutralized by adding 0.125 volumes of 0.1 N NaOH on ice, followed by the addition of the remainder of components. Next organoids, resuspended in the appropriate culture media, were added to the solution. The hydrogels were polymerized in 4-chamber slides (Corning) at 37 C and 5% CO2 for 1 hr, at which point culture media was added and the gels were detached from the slide with a pipette tip.

***Hormone Treatment***

When indicated, hormones were added directly to the ECM hydrogels during fabrication, and maintained in the culture medium; compounds added after hydrogel fabrication were maintained in the culture medium. β-estradiol (Sigma Aldrich) was added at a concentration of 10ng/ml, progesterone (Sigma Aldrich) at 500ng/ml, prolactin (Sigma Aldrich) at 1µg/ml, and BPE (Lonza) at 0.3%.

***Lentiviral Production***

Lentivirus production was performed as previously described . LeGO lentiviral vectors were kindly provided by Kristoffer Riecken . Virus was produced from three separate vectors encoding mCherry, Venus, and Cerulean fluorescent proteins.

***Immunofluorescence/Immunohistochemistry***

Samples were fixed with 4% paraformaldehyde for 30 minutes at room temperature. Pads were permeabilized overnight using 0.1% TritonX-100 and incubated with blocking solution (PBST with 3% goat serum and 3% BSA) for 2 hr at room temperature and stained with the appropriate primary antibody in blocking buffer overnight at 4⁰C. The samples were washed with PBS, and incubated with an Alexa Fluor-labeled secondary antibody (Cell Signaling) and phalloidin-647 (Life Technologies). Samples were washed, stained with 1ug/ml DAPI.

BrdU (Sigma Aldrich) was added at 10 µM for 2 hr, after which samples were washed with PBS and fixed with 4% paraformaldehyde. Anti-BrdU antibody was purchased from Cell Signaling Technologies and staining was performed according to manufacturer protocol.

IHC embedding, sectioning, and staining was performed at the Koch Institute Histology Core Facility. Samples were fixed in 4% neutral buffered formalin overnight, washed with 70% ethanol, and paraffin embedded. IHC was performed using the ThermoScientific IHC Autostainer 360.

Primary antibodies used in this study for IF were CK14 (Life Technologies; 1:300; RB-9020-P), CK8/18 (Vector; 1:500; VP-C407), SLUG (Cell Signaling; 1:400;C19G7), and SOX9 (Sigma; 1:50; WH0006662M2). IHC antibodies used were GATA3 (Cell Signaling; 1:6400; 5852), MUC1 (AbCAM; 1:100; 15481).

***Microscopy***

Immunofluorescence images were captured using a Zeiss LSM 700 and analyzed with LSM Viewer. IHC images were captured using Zeiss Axiophot. Time-lapse movies were captured using a Nikon TE2000 with a heated stage and 5% CO2.

***Physical characterization of hydrogels***

The elastic modulus of the hydrogels was measured via Hertzian analysis of atomic force microscopy (AFM) force curves . Hydrogels were mounted on a glass slide and placed in the AFM (Veeco, Nanoscope IV with picoforce scanner head). The tip (Novascan, k=14N/m functionalized with 45 µm polystyrene ball) was then brought into contact with the sample. Force displacement curves were obtained by monitoring the deflection of the tip throughout a z-displacement of 4 microns. Force exhibited by on the tip was calculated according to the equation:


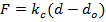


where k_c_ is the spring constant of the cantilever (14 N/m), d-d_o_ is the tip deflection following contact with the gel.

According to Hertzian analysis, the modulus may then be determined by the following equation, which accounts for the spherical geometry of the AFM tip:


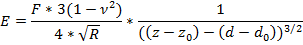


Where (z-z_0_) is the applied translation of the cantilever, r is the radius of the tip, and ν is the Poisson’s ratio (assumed to be 0.5 for an incompressible hydrogel). The elastic modulus was calculated using a linear regression of the force on the tip and the displacement relative to the gel, and then correcting for the geometric factors corresponding to tip geometry and Poisson’s ratio.

Swelling ratios (SR) were calculated using the equation:


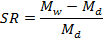


where M_w_ = wet weight and M_d_ = dry weight.
