# Supplementary material for: Growth of human breast tissues from patient cells in 3D hydrogel scaffolds
Source: Breast Cancer Res. 2016 Mar 1;18:19. doi: 10.1186/s13058-016-0677-5 (PMC4772689; doi:10.1186/s13058-016-0677-5)

Figure S1

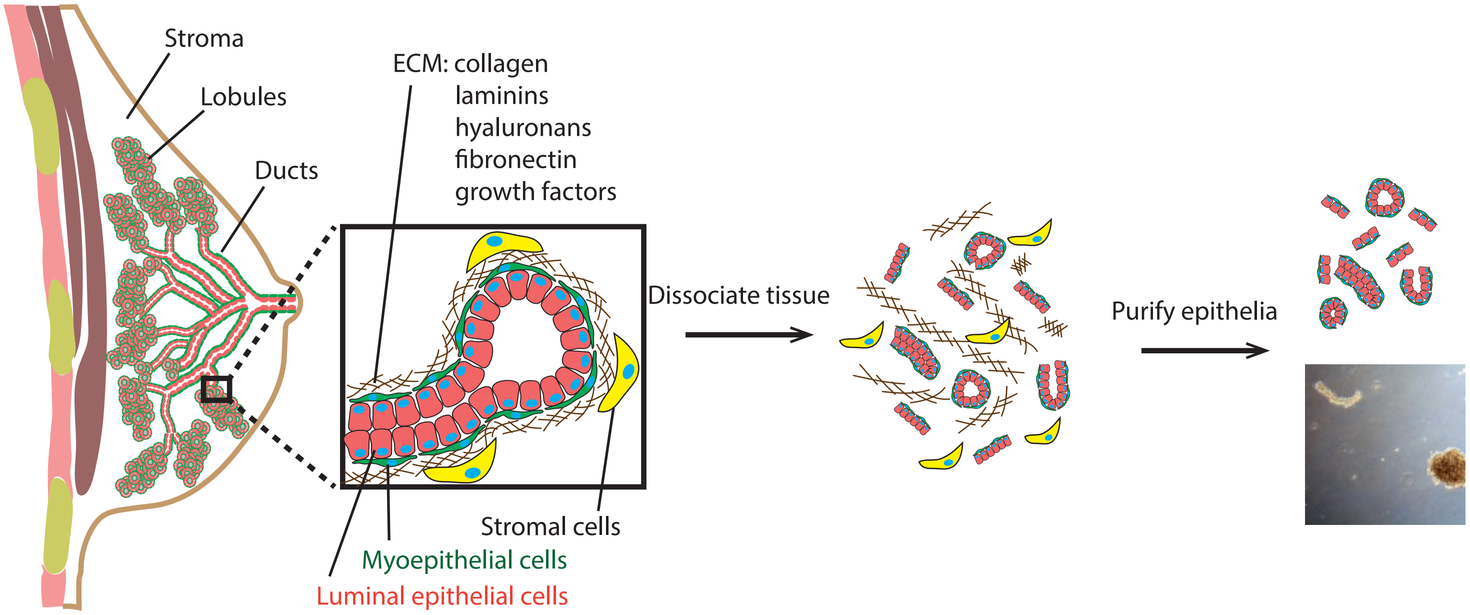
Stroma
Lobules
Ducts
ECM: collagen
laminins
hyaluronans
fibronectin
growth factors
Stromal cells
Myoepithelial cells
Luminal epithelial cells
Dissociate tissue
Purify epithelia

Figure S2

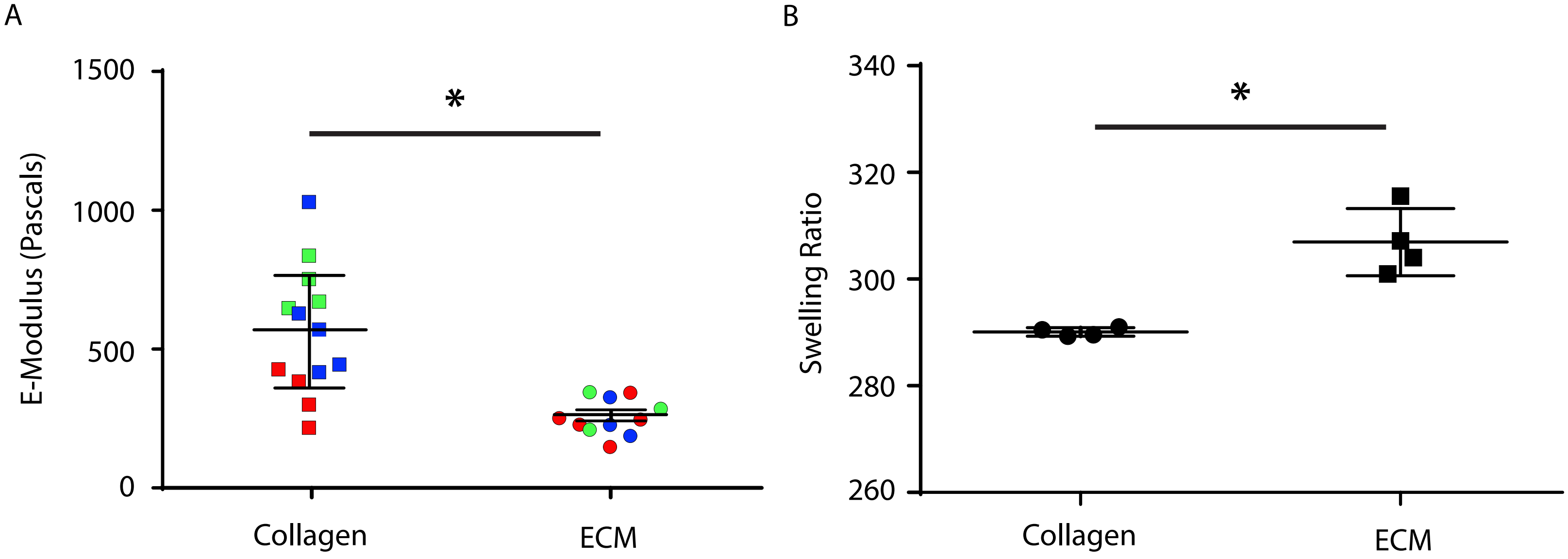

A
1500
1000
500
0
E-Modulus (Pascals)
*
Collagen
ECM
B
340
320
300
280
260
Swelling Ratio
*
Collagen
ECM

Figure S3

A

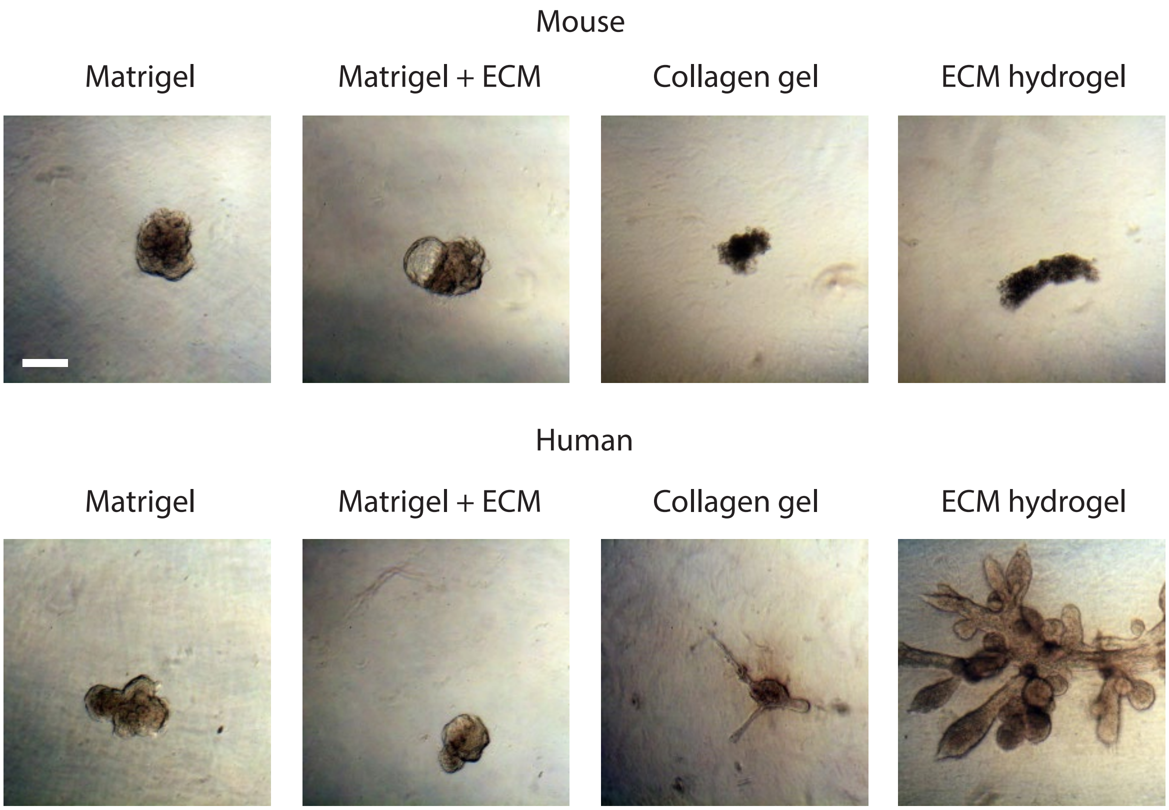
Mouse
Matrigel
Matrigel + ECM
Collagen gel
ECM hydrogel
Human
Matrigel
Matrigel + ECM
Collagen gel
ECM hydrogel

B

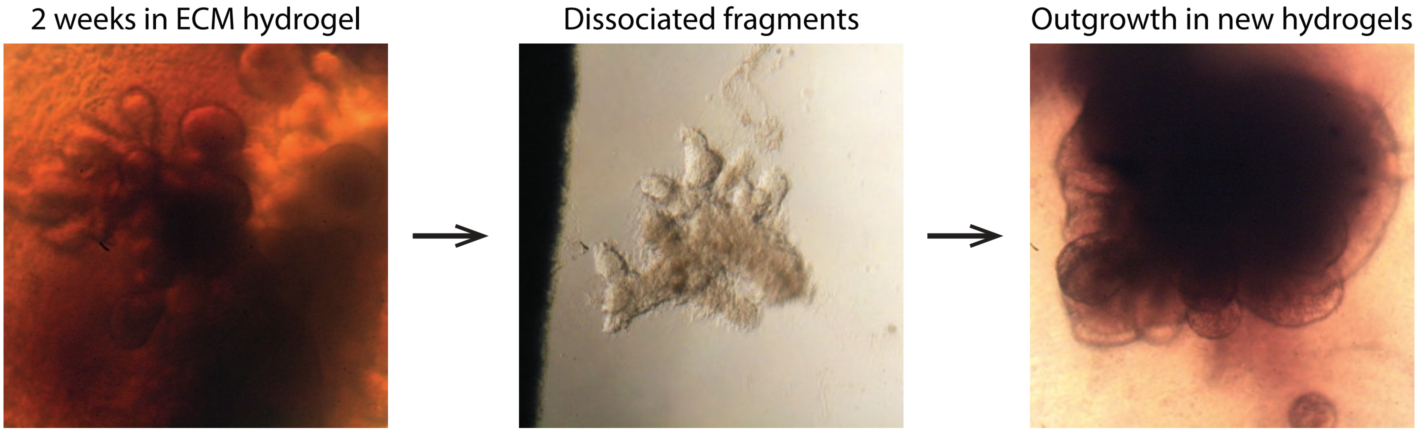
2 weeks in ECM hydrogel
Dissociated fragments
Outgrowth in new hydrogels

Figure S4

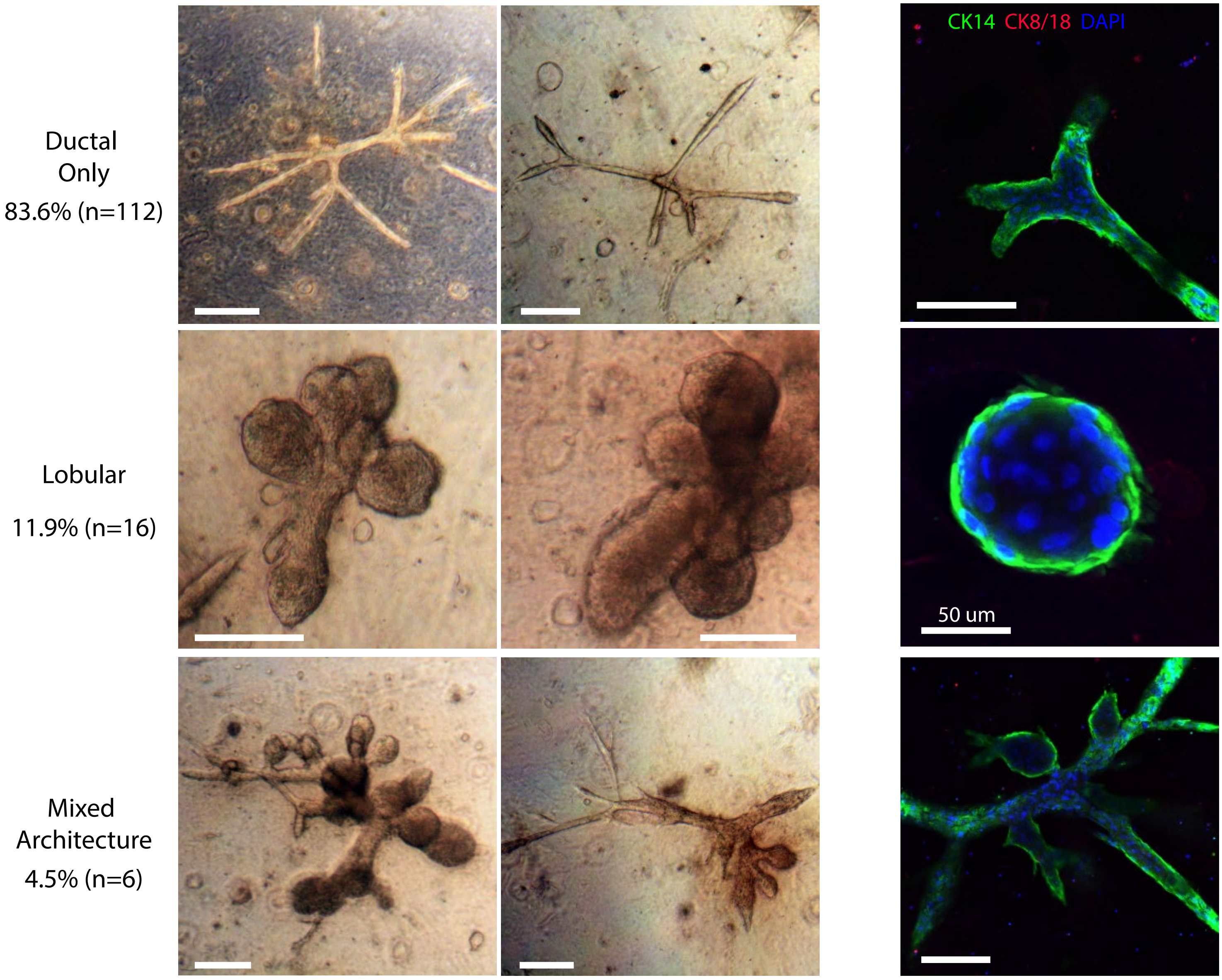

Ductal
Only
83.6% (n=112)
Lobular
11.9% (n=16)
Mixed
Architecture
4.5% (n=6)
CK14 CK8/18 DAPI
50 um

Figure S5

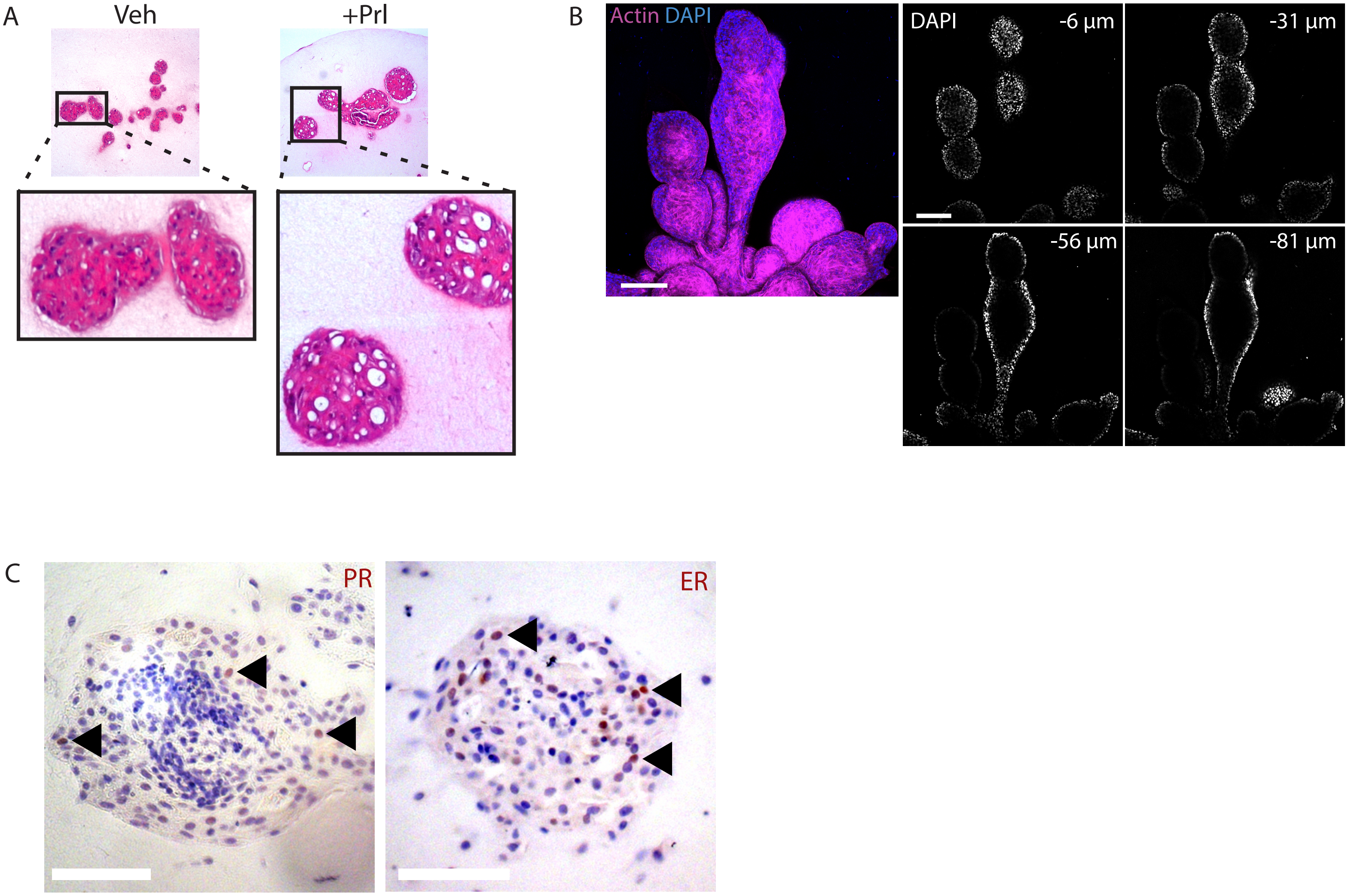

Figure S6

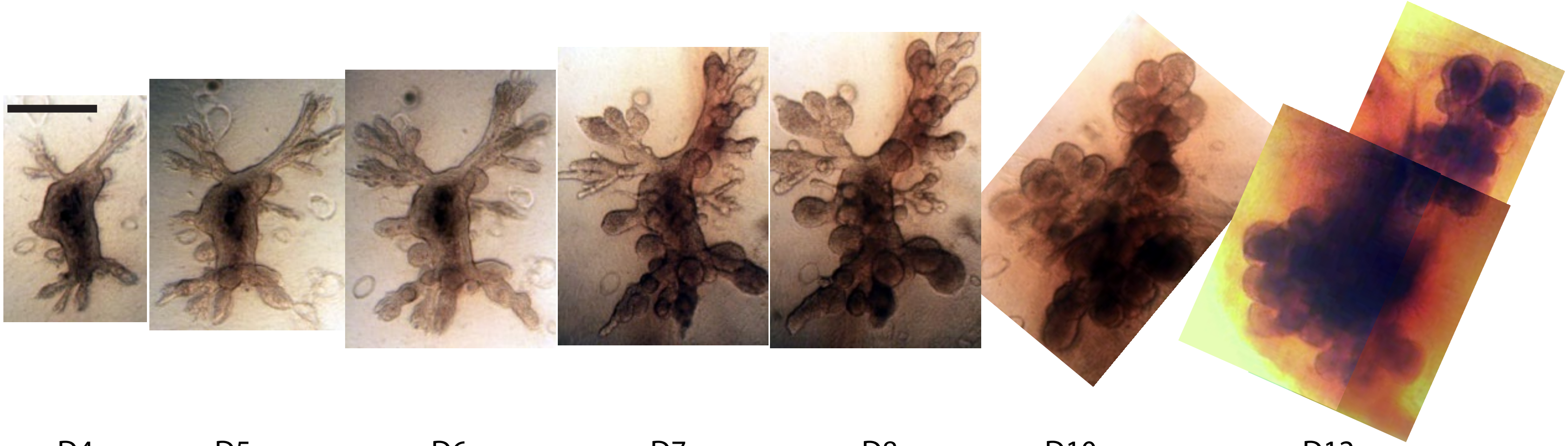

D4 D5 D6 D7 D8 D10 D12

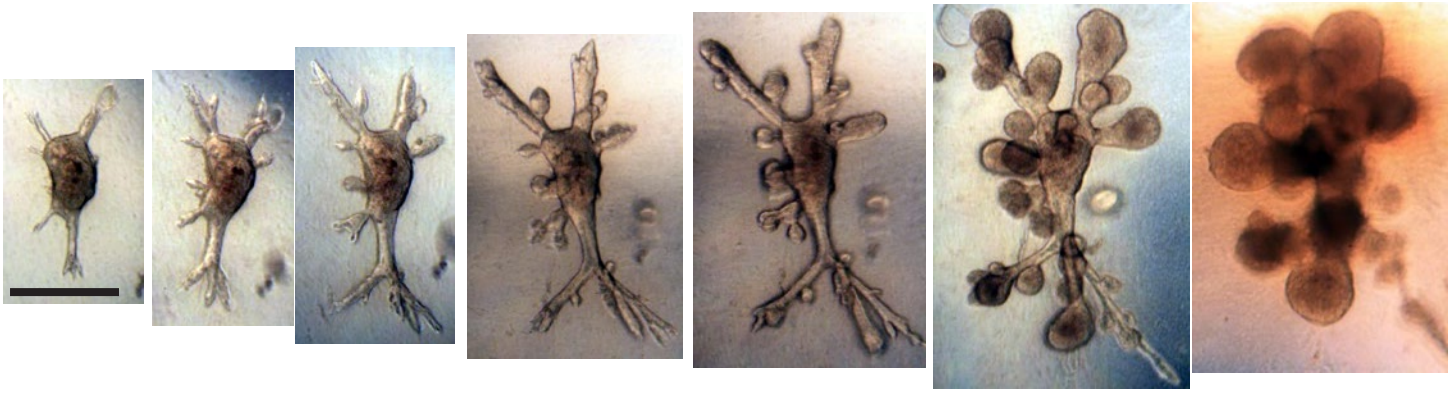

D4 D5 D6 D7 D8 D10 D12

Figure S7

A

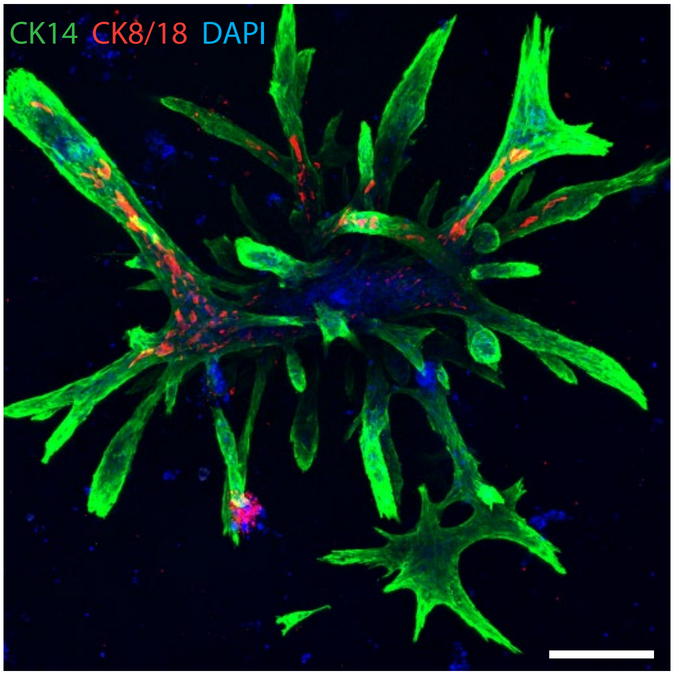

B

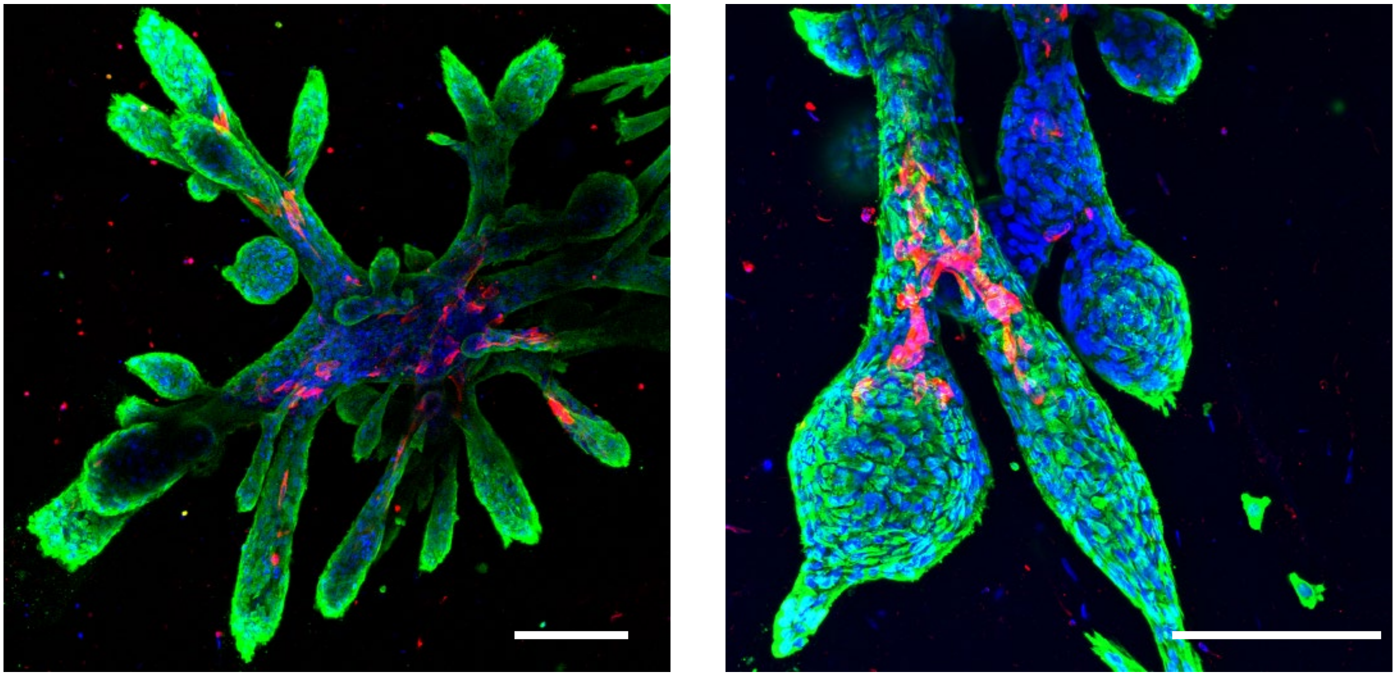

Figure S8

A

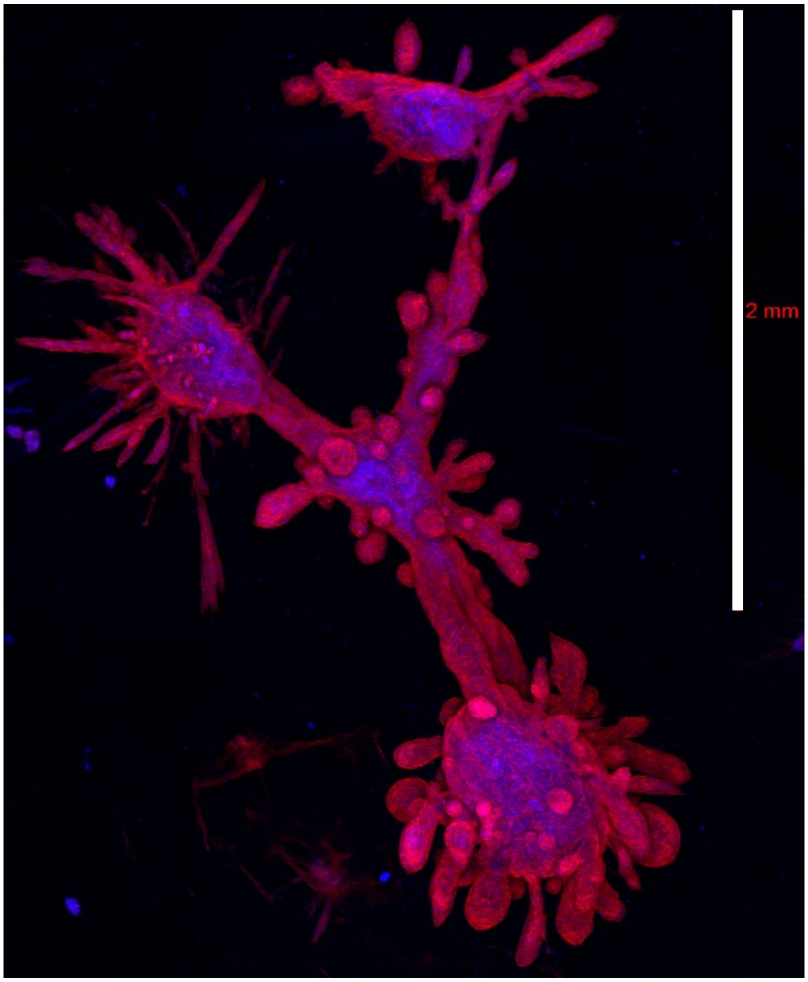

B

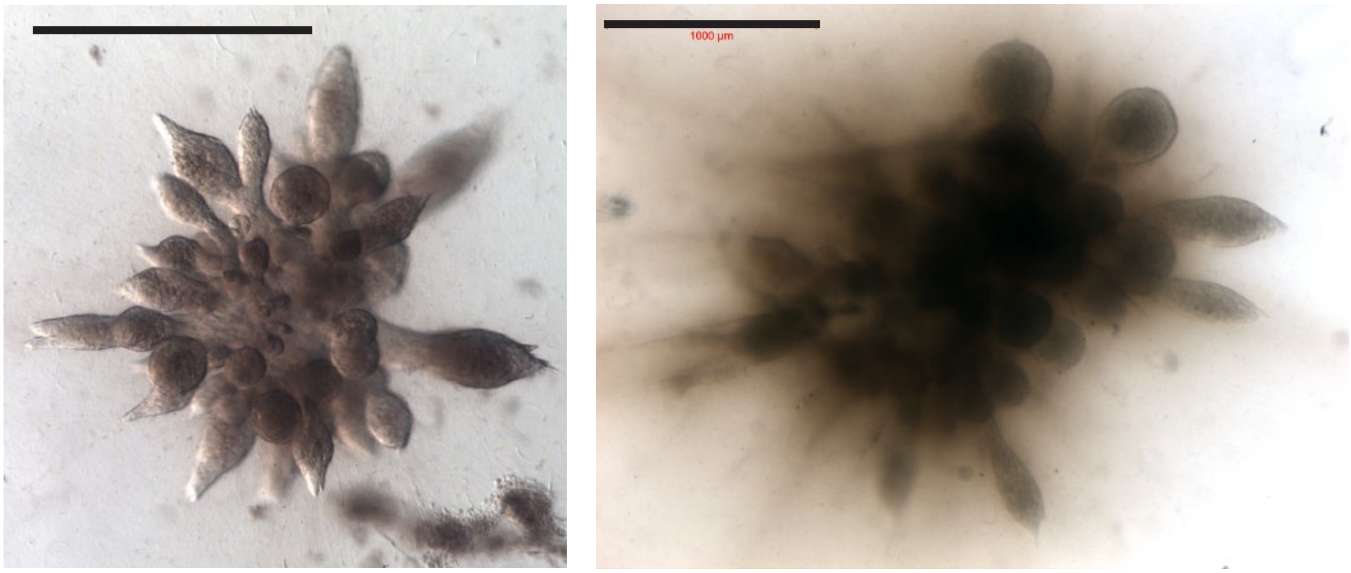

Figure S9

A

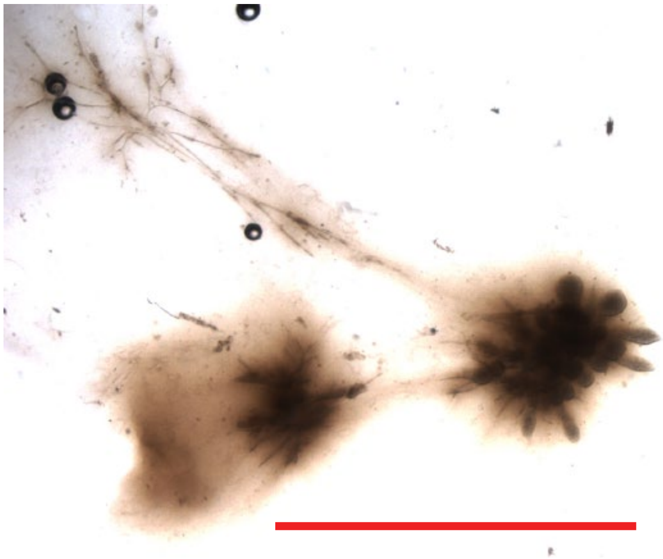

B

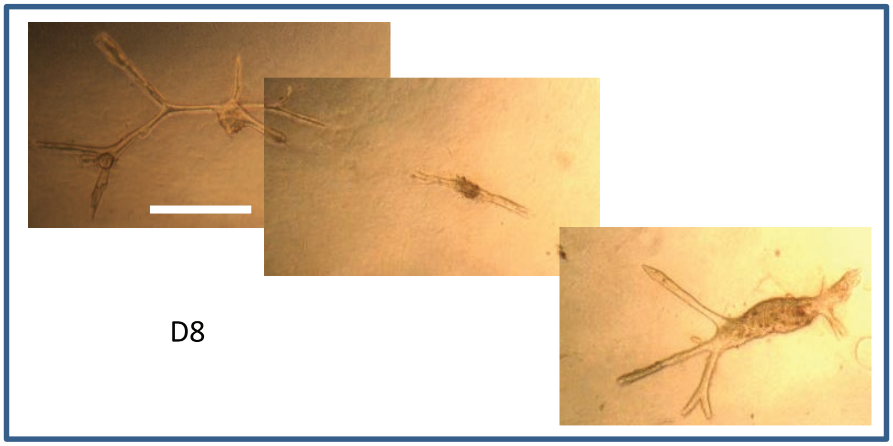

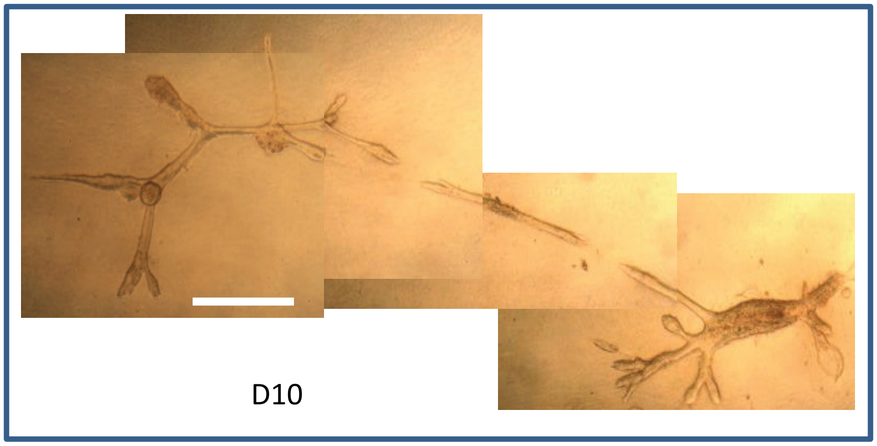

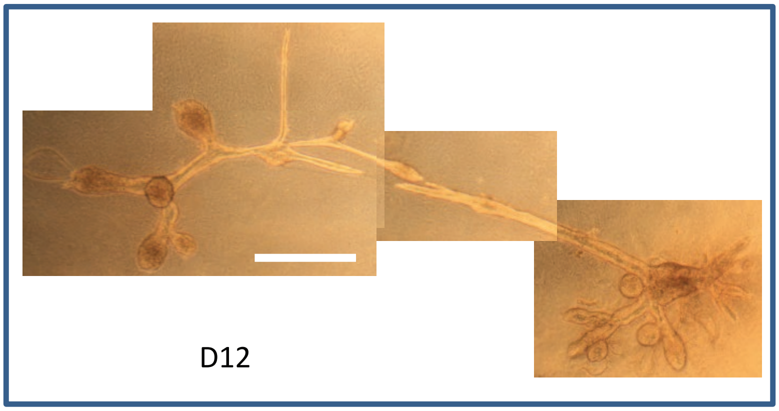

Supplement: Additional file 2: Supplemental Figures. — Figure S1 Schematic for the dissociation of reduction mammoplasty tissue. Schematic representation of tissue dissociation and purification of epithelium. For more details, see Additional file 1: Supporting Methods. Figure S2 Physical characterization of collagen and ECM hydrogels. a Young’s modulus was measured at least three times for each of three independent replicates (red, blue, and green) for collagen gels and ECM hydrogels. Plotted are the mean and standard deviation for the replicates. b The swelling ratio was calculated for collagen gels and ECM hydrogels for four independent replicates. Plotted are the mean and standard deviation. *p < 0.05. Figure S3 Comparison of various 3D scaffolds seeded with mouse or human mammary tissue. Representative bright-field images of human or mouse mammary epithelial tissue fragments grown for 10 days in either Matrigel alone (Matrigel); Matrigel supplemented with fibronectin, laminins, hyaluronans, insulin, epidermal growth factor, and hydrocortisone (Matrigel + ECM); collagen hydrogels (collagen gel); or collagen hydrogels supplemented with fibronectin, laminins, hyaluronans, insulin, EGF, and hydrocortisone (ECM hydrogel). b Representative bright-field images of an organoid grown in an ECM hydrogel (left), removed from the primary gel using collagenase treatment and fragmented (middle), and producing new outgrowths after being passaged into a secondary ECM hydrogel (right). Scale bars represent 200 μm. Figure S4 Single mammary epithelial cells produce heterogeneous structure morphologies in hydrogels. Representative bright-field and immunofluorescence images of structures formed from single cells after 18 days of growth in hydrogels. These structures are highly heterogeneous but generally fall into three classes: ductal structures with narrow ducts and no lobules (top), lobular structures with short and wide ducts (middle), and structures with mixed ductal and lobular architecture (bottom). The majority of structures form [file 13058_2016_677_MOESM2_ESM.pdf]
